# Supplementary material for: APOE3 astrocytes can rescue lipid abnormalities and dystrophic neurites of APOE4 human neurons
Source: bioRxiv. 2025 Oct 24:2025.10.24.684364. Preprint. [Version 1] doi: 10.1101/2025.10.24.684364 (PMC12633476; doi:10.1101/2025.10.24.684364)
Supplement: 1 [file NIHPP2025.10.24.684364V1-supplement-1.pdf]

bioRxiv preprint doi: https://doi.org/10.1101/2025.10.24.684364; this version posted October 24, 2025. The copyright holder for this preprint (which was not certified by peer review) is the author/funder, who has granted bioRxiv a license to display the preprint in perpetuity. It is made available under aCC-BY 4.0 International license.

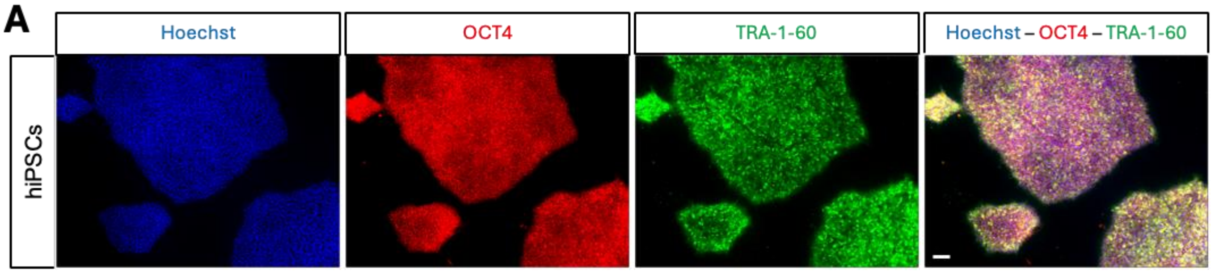

B

APOE isoform	rs429358	rs7412
E2	TGC	TGC
E3	TGC	CGC
E4	CGC	CGC

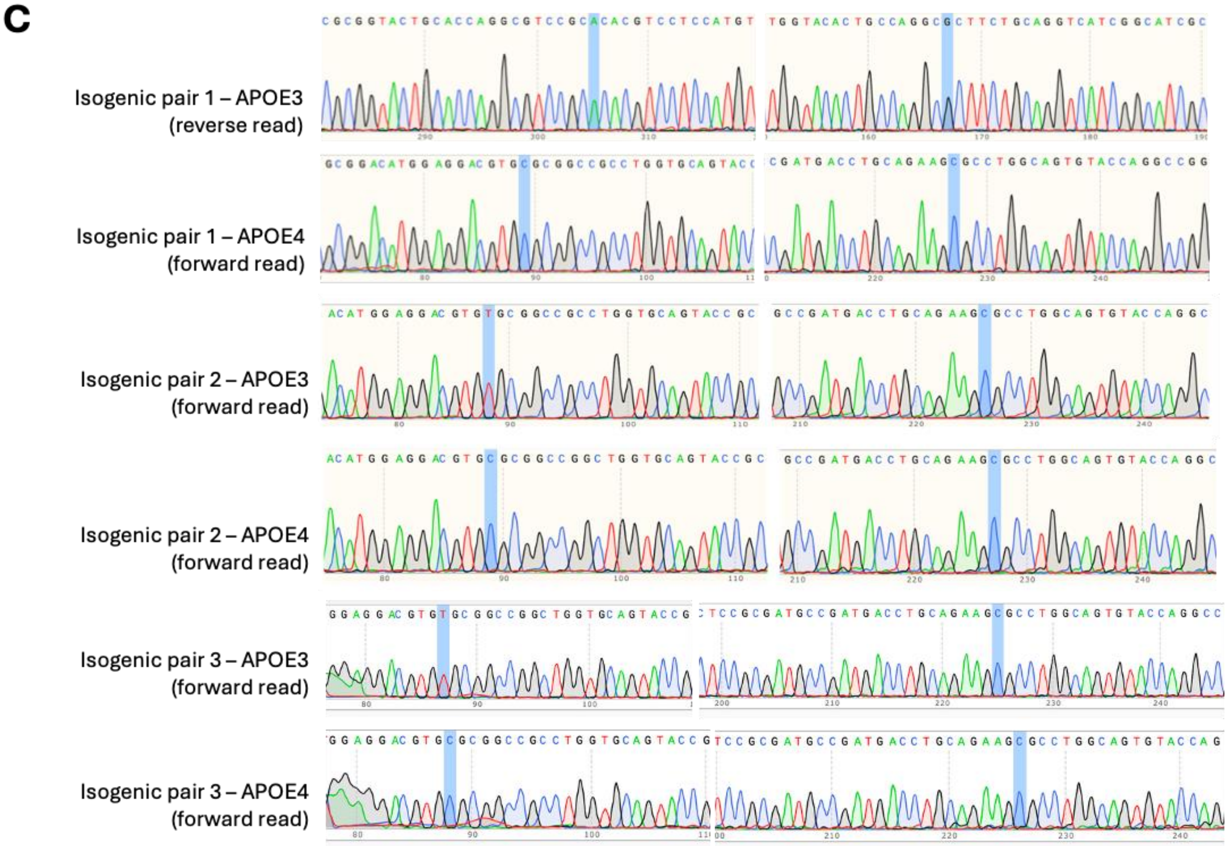

Figure S1. APOE genotyping of isogenic hiPSC pairs used in this study.
(A) hiPSCs expressing pluripotency markers OCT4 (red) and TRA-1-60 (green). Nuclei were stained with Hoechst (blue). Scale bar: 100 µm.
(B) The two single nucleotide polymorphisms (SNPs) that distinguish the 3 APOE isoforms are shown: rs429358 (T>C) and rs7412 (C>T).

bioRxiv preprint doi: https://doi.org/10.1101/2025.10.24.684364; this version posted October 24, 2025. The copyright holder for this preprint (which was not certified by peer review) is the author/funder, who has granted bioRxiv a license to display the preprint in perpetuity. It is made available under aCC-BY 4.0 International license.

(C) Sanger sequencing confirms the presence of APOE3 or APOE4 SNPs in 3 isogenic hiPSC pairs.

bioRxiv preprint doi: https://doi.org/10.1101/2025.10.24.684364; this version posted October 24, 2025. The copyright holder for this preprint (which was not certified by peer review) is the author/funder, who has granted bioRxiv a license to display the preprint in perpetuity. It is made available under aCC-BY 4.0 International license.

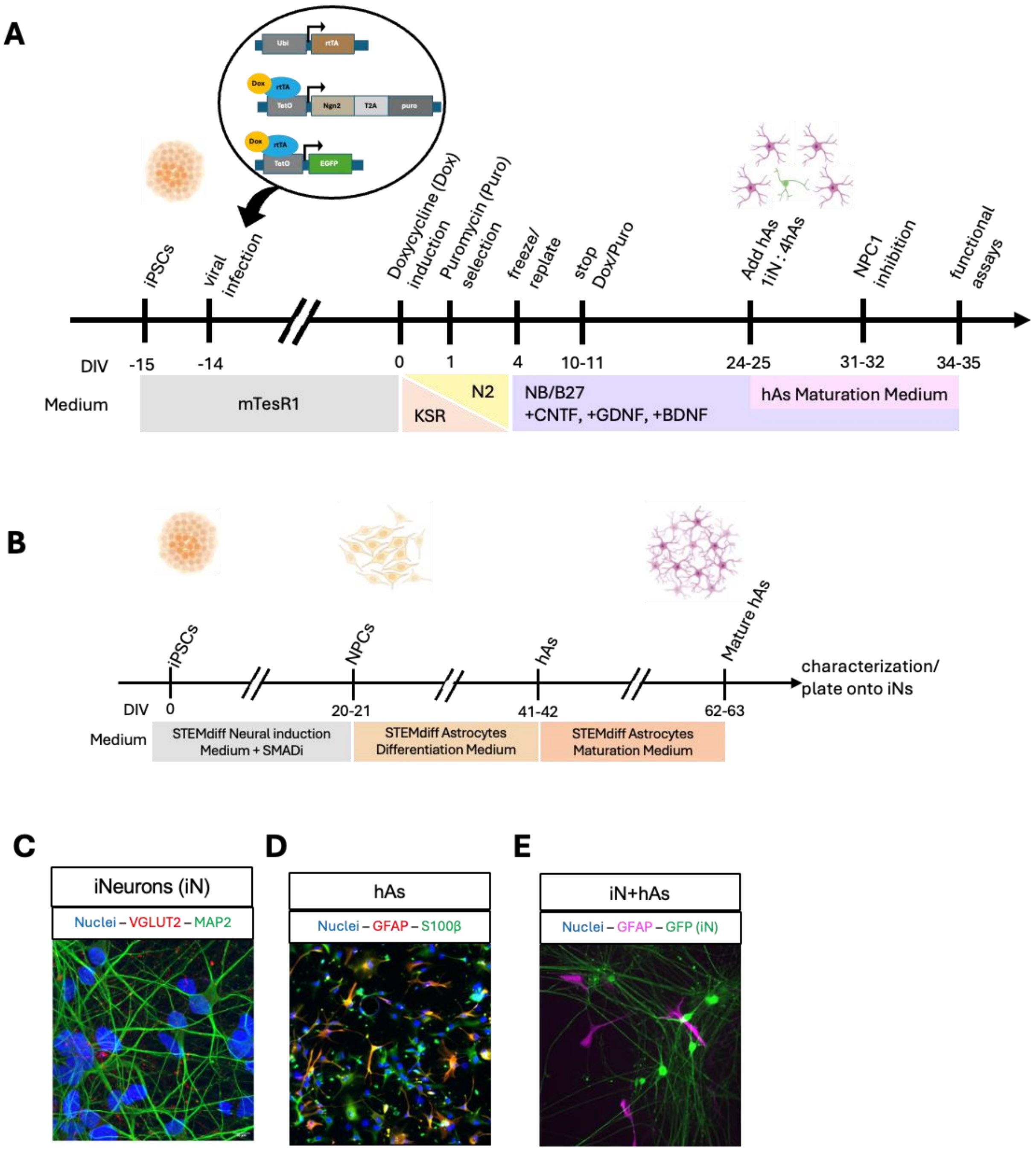

Figure S2. Generation of APOE3 and APOE4 iN and hAs from hiPSCs.
(A) Schematic of neuronal differentiation. Excitatory glutamatergic iNeurons (iN) were generated from APOE3 or APOE4 hiPSCs by lentiviral transduction of NGN2 and GFP. NGN2 expression was induced with doxycycline, and transduced cells were selected with puromycin. Following 2 weeks of differentiation, iN were cocultured with astrocytes for 1 week and subsequently treated with the NPC1 inhibitor for 3 days to induce lipid stress.
(B) Schematic of astrocyte differentiation. APOE3 or APOE4 hiPSCs were first differentiated into neural progenitor cells (NPCs) using STEMdiff™ Neural Induction Medium

bioRxiv preprint doi: https://doi.org/10.1101/2025.10.24.684364; this version posted October 24, 2025. The copyright holder for this preprint (which was not certified by peer review) is the author/funder, who has granted bioRxiv a license to display the preprint in perpetuity. It is made available under aCC-BY 4.0 International license.

supplemented with SMAD inhibitors. NPCs were then cultured in STEMdiff™ Astrocyte Medium for 3 weeks, followed by an additional 3 weeks in STEMdiff™ Astrocyte Maturation Medium to obtain mature astrocytes (hAs).
(C-E) Representative immunofluorescence images of cell type-specific markers. (C) Excitatory glutamatergic iN labeled with VGLUT2 (red) and the pan-neuronal marker MAP2 (green), (D) Mature hAs expressing astrocyte markers GFAP (red) and S100β (green), (E) iN+hAs cocultures, where GFP (green) marks iN, and GFAP (magenta) marks hAs.
